# Peroneus Longus Autograft Harvest and Preparation for Anterior Cruciate Ligament Reconstruction

**DOI:** 10.1002/atn2.70255

**Published:** 2026-09-11

**Authors:** Mathew Park, Nathan Bouwkamp, Thomas Riggs, Kendall Hamilton, Travis Menge

**Affiliations:** ^1^ Department of Orthopaedic Surgery Corewell Health West Grand Rapids Michigan U.S.A.; ^2^ College of Human Medicine Michigan State University East Lansing Michigan U.S.A.

## Abstract

The peroneus longus tendon (PLT) has emerged as a promising autograft option for anterior cruciate ligament reconstruction because of its favorable biomechanical properties, consistent graft diameter, and minimal donor‐site morbidity; however, detailed descriptions of graft harvesting and preparation techniques remain limited within literature. This report aims to construct a reproducible step‐by‐step method for harvesting and preparing a PLT autograft with internal brace augmentation. This technical report offers a detailed description with the goal of standardizing these operative steps that may facilitate broader adoption of PLT autograft and support ongoing investigation into PLT autografts as an alternative option for anterior cruciate ligament reconstruction.

## Graphical abstract

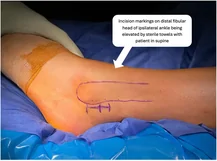

**VIDEO 1:** We begin by identifying and marking our key landmark, the distal fibula which the peroneal tendons lie posterior too. The patient is lying supine with a small stack of folded sterile towels under the foot to help make the harvest site easily accessible. A 2‐ to 3‐cm incision is made just proximal to the tip of the distal fibula, about 1 cm posterior to its border. The soft tissue is then dissected, and the fascia is sharply opened, revealing the more superficial peroneus longus and the deeper peroneus brevis. A curved hemostat is then passed deep to the peroneus brevis, allowing us suture peroneus longus to the peroneus brevis using a #0 FiberWire for tenodesis. This is followed by a running 1.3‐mm FiberLoop SutureTape whipstitch which is placed in the peroneus longus, beginning 3 to 4 cm proximal to the tenodesis and working distally. This stitch will serve as our control during harvest. The longus is then transected proximal to the tenodesis and distal to the whipstitch. The sutured end is passed into a tendon stripper and advanced proximally, keeping the stripper parallel to the fibula. The tendon is harvested, taking care to stop at least 5 cm distal to the fibular head to avoid injury to the deep peroneal nerve. Once harvested, the tendon is placed onto a graft preparation board for measurement. In our case, the tendon measured approximately 270 mm. The graft is then tripled over onto itself to achieve a final length of 75 to 80 mm, improving structural strength and reducing elongation under load. After tripling, the diameter is sized as 10 mm and is confirmed using a graft sizing block. We then move into graft end preparation. The tibial side is secured using a number 1.3 FiberLoop suture tape with the SpeedWhip technique, incorporating about 20 mm of graft for fixation. The femoral end is bullet‐tapered to allow smooth passage into a 10‐mm femoral tunnel. An Arthrex FiberTag TightRope II Implant with InternalBrace is attached for suspensory fixation, again incorporating around 20 mm of tendon. The original Arthrex InternalBrace component is removed and replaced with a Zimmer ActivBraid Collagen Co‐Braid to provide additional collagen to the graft construct as a biointegrative high‐strength collagen suture. The Arthrex InternalBrace or the Zimmer ActivBraid Collagen Co‐Braid can be used based on surgeon preference. Seating marks are then applied to guide femoral button positioning. A mark is placed 38 mm from the button to match cortical thickness, and a second mark 20 mm from the femoral graft end to confirm full seating into the femoral socket during fixation, completing our peroneus longus tendon autograft construct. Video content can be viewed at https://doi.org/10.1002/atn2.70255.

Anterior cruciate ligament (ACL) injuries are among the most common ligamentous knee injuries and frequently require surgical reconstruction to restore stability, prevent recurrent episodes of instability, and reduce the long‐term risk of secondary joint damage. Autograft selection plays a central role in achieving successful outcomes, with bone–patellar tendon–bone, hamstring tendon, and quadriceps tendon grafts serving as the standard options.^1^ Although these grafts have proven effective, concerns such as donor‐site discomfort, muscle weakness, variable graft size, and patient‐specific anatomical limitations have prompted continued exploration of alternative graft sources.^2^ Within this context, the peroneus longus tendon (PLT) has gained attention for its consistent diameter, high tensile strength, and minimal donor‐site morbidity, making it a compelling option when traditional autografts are unsuitable or suboptimal.^3^, ^4^, ^5^

As the use of the PLT expands, an equally important consideration is the precision of harvesting and graft preparation techniques, which directly influence reconstruction quality and postoperative performance. The addition of internal brace augmentation, regardless of the graft type, offers additional mechanical support during the early phases of healing, potentially improving durability and functional recovery.^6^ However, despite growing clinical interest, the literature still lacks standardized descriptions of the operative steps involved in harvesting and preparing this graft. Therefore, the objective of this project is to provide a comprehensive, step‐by‐step account of the PLT harvest and preparation technique to enhance reproducibility and support broader adoption of this evolving approach to ACL reconstruction.

## SURGICAL TECHNIQUE

**FIGURE 1:**
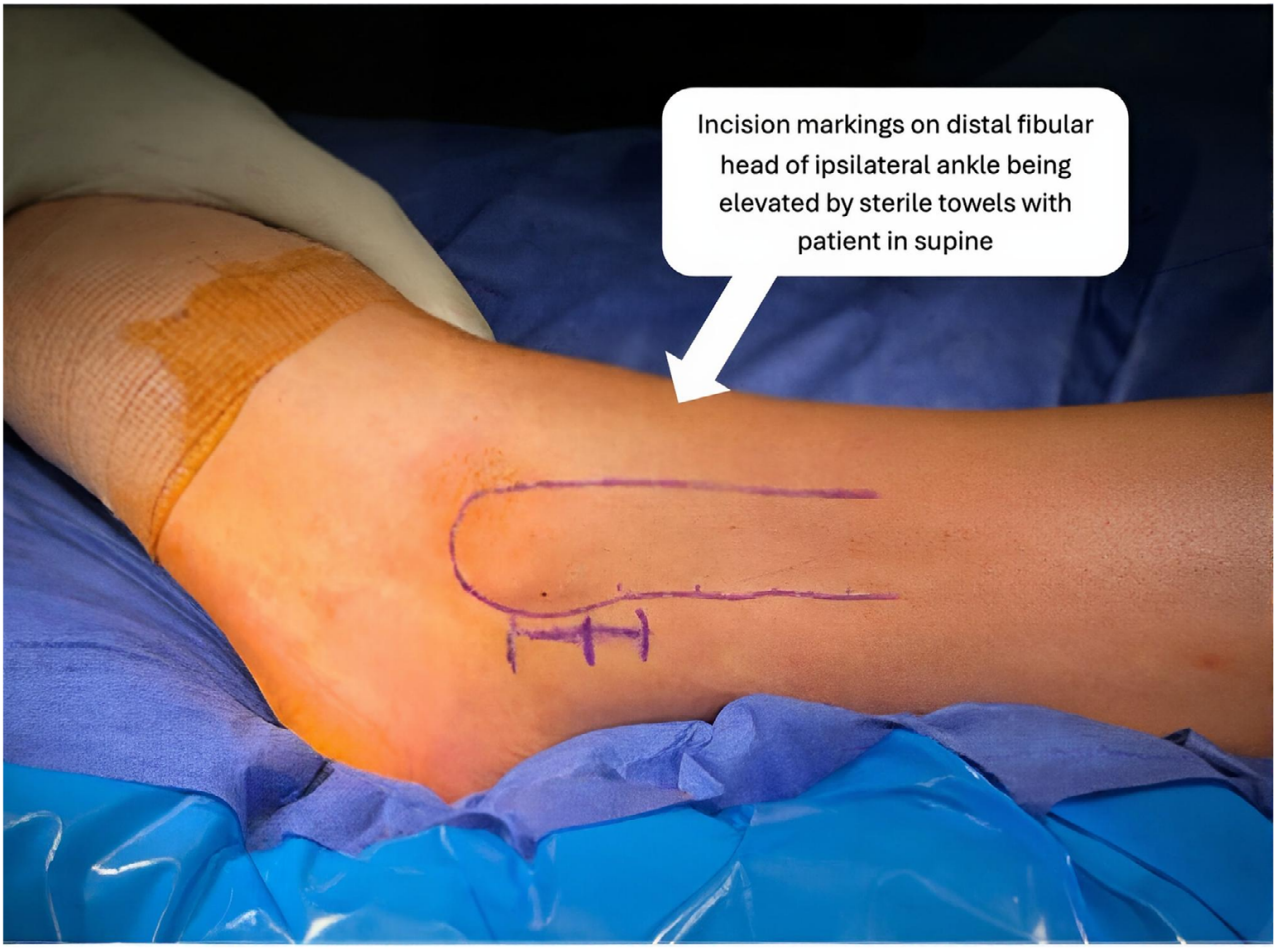
Peroneus longus tendon graft harvesting markings. The image shows peroneus longus graft autograft harvest preparation from the patient's ipsilateral ankle, with the patient lying supine and a small stack of folded sterile towels under the foot for easier harvest site access, as it is marked for surgical incision. Markings were made around the distal fibular head as the peroneus tendons run just posterior to it. These markings will help guide the initial 2‐ to 3‐cm incision that run proximal to the distal fibular head.

**FIGURE 2:**
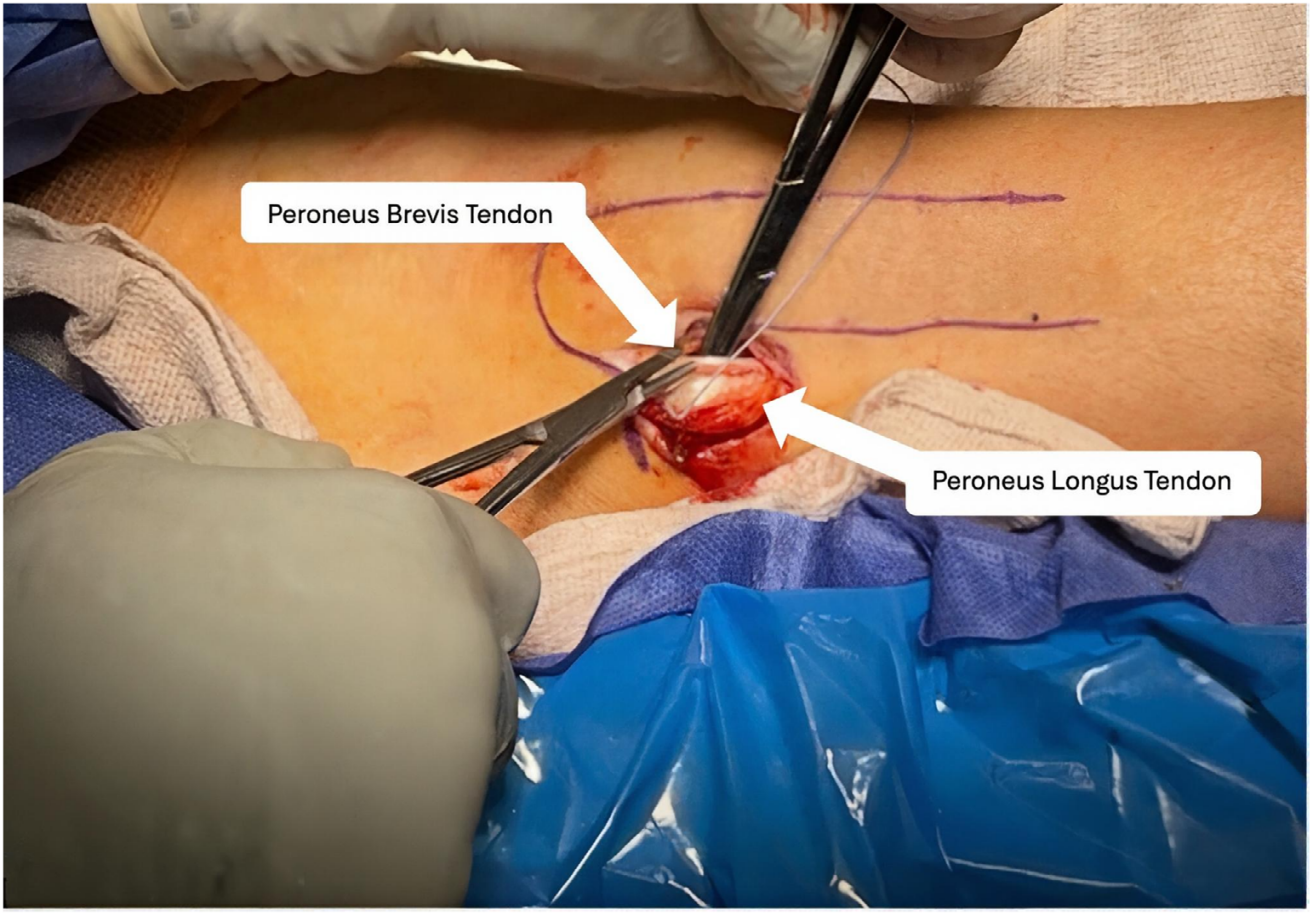
Tenodesis of peroneus longus and peroneus brevis tendon. The peroneus longus and brevis tendon is shown being brought outside of the skin via an Acufex Smith and Nephew Curved Hemostat (Acufex Smith and Nephew) for suture tenodesis of longus to brevis with a #0 FiberWire Suture (Arthrex) proximal to the tip of the distal fibula. When brought outside of the skin as shown in this image, the peroneus brevis tendon is at the top whereas the bottom being shown in the image is the peroneus longus tendon.

**FIGURE 3:**
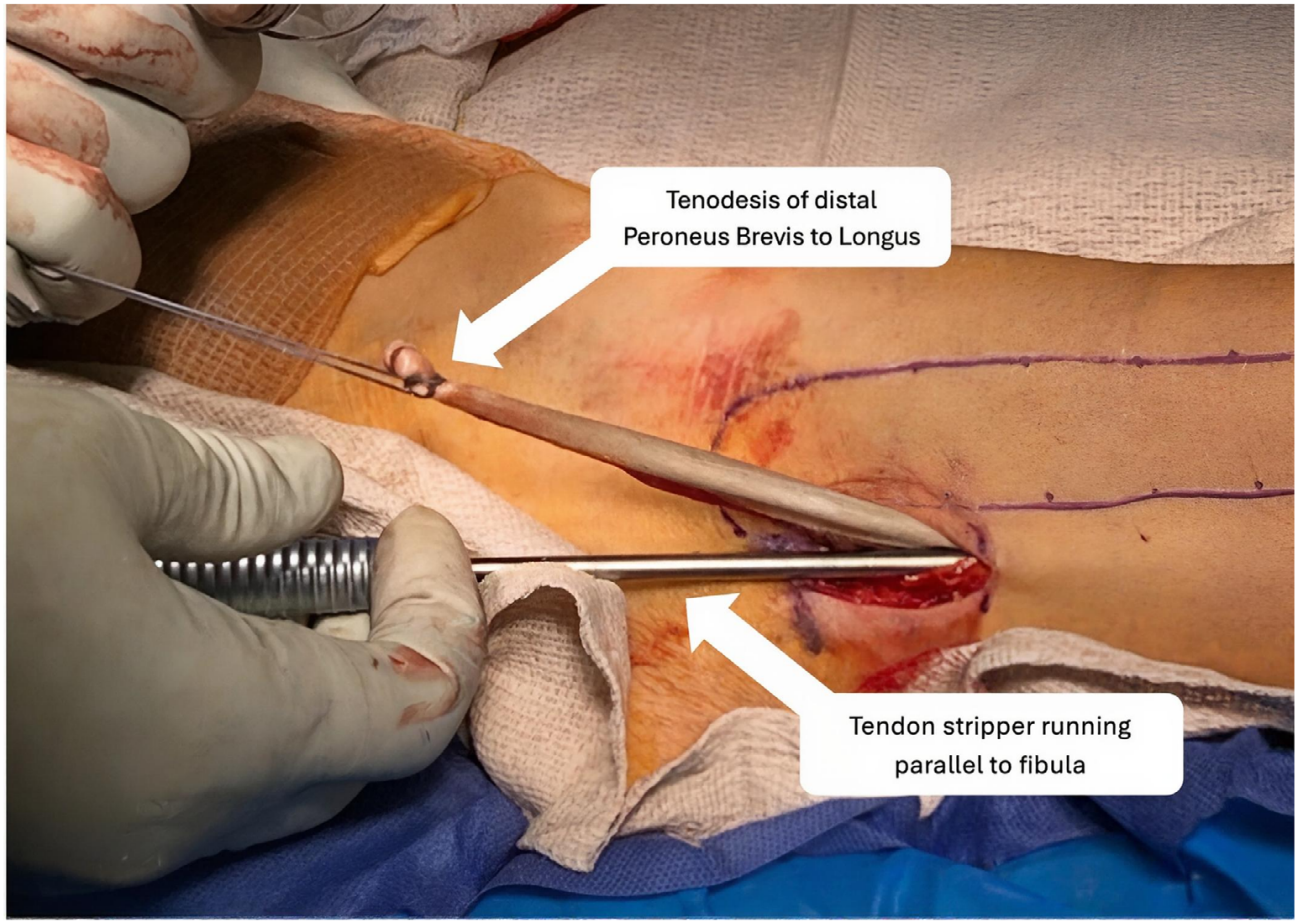
Transected end of peroneus longus with whipstitch and tendon stripper placement. The distally transected end of the peroneus longus is shown, controlled by the 1.3‐mm FiberLoop SutureTape whipstitch (Arthrex) sutures and is placed through a Tendon Stripper Open (Acufex Smith and Nephew) for proximal resection parallel to the fibula. The Tendon Stripper Open (Acufex Smith and Nephew) will run parallel to the fibula until it reaches 5 cm distal to the proximal fibular head.

**FIGURE 4:**
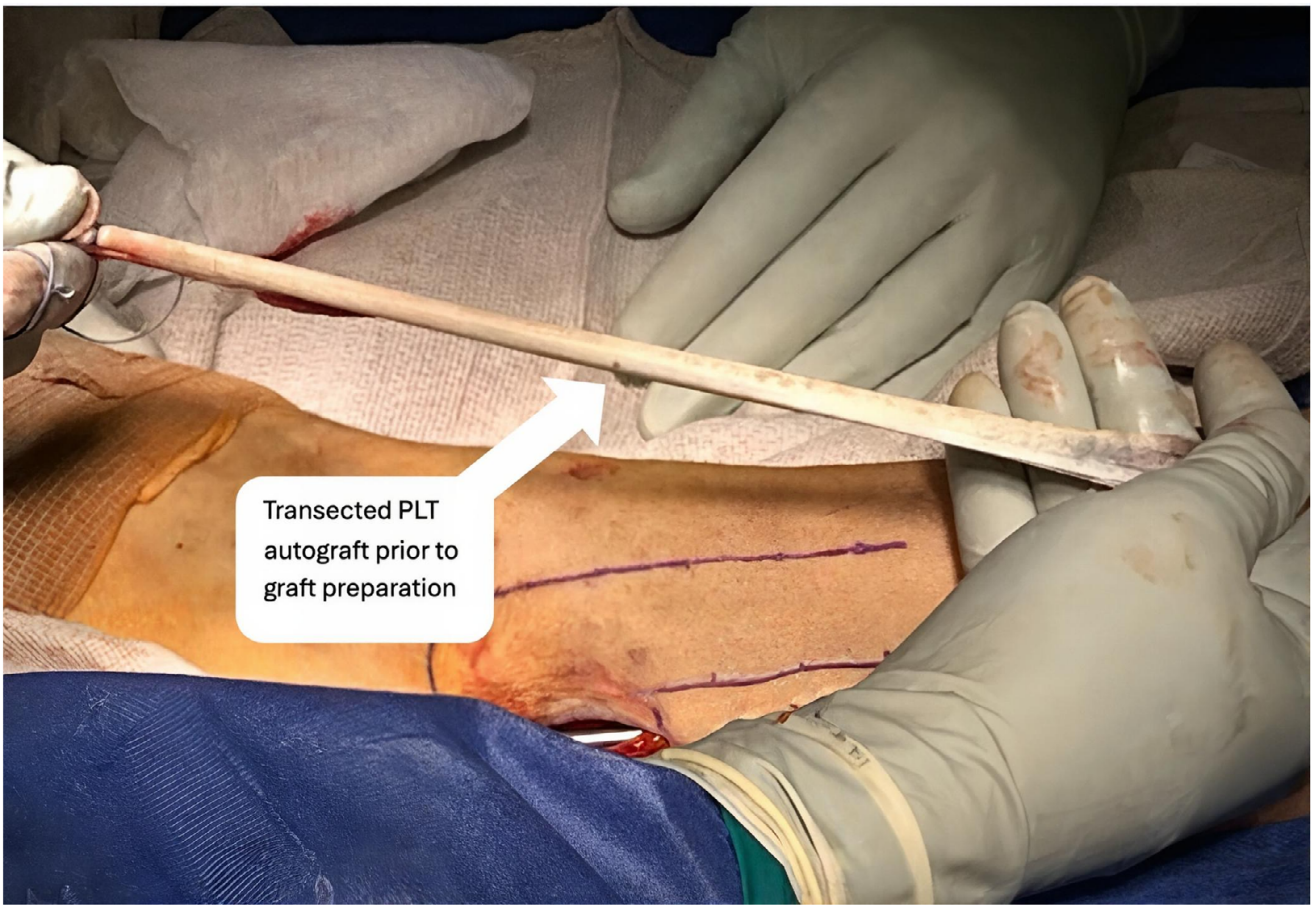
Complete resection of peroneus longus autograft. This figure shows full resection of the PLT that is now ready for graft preparation. (PLT, peroneus longus tendon.)

**FIGURE 5:**
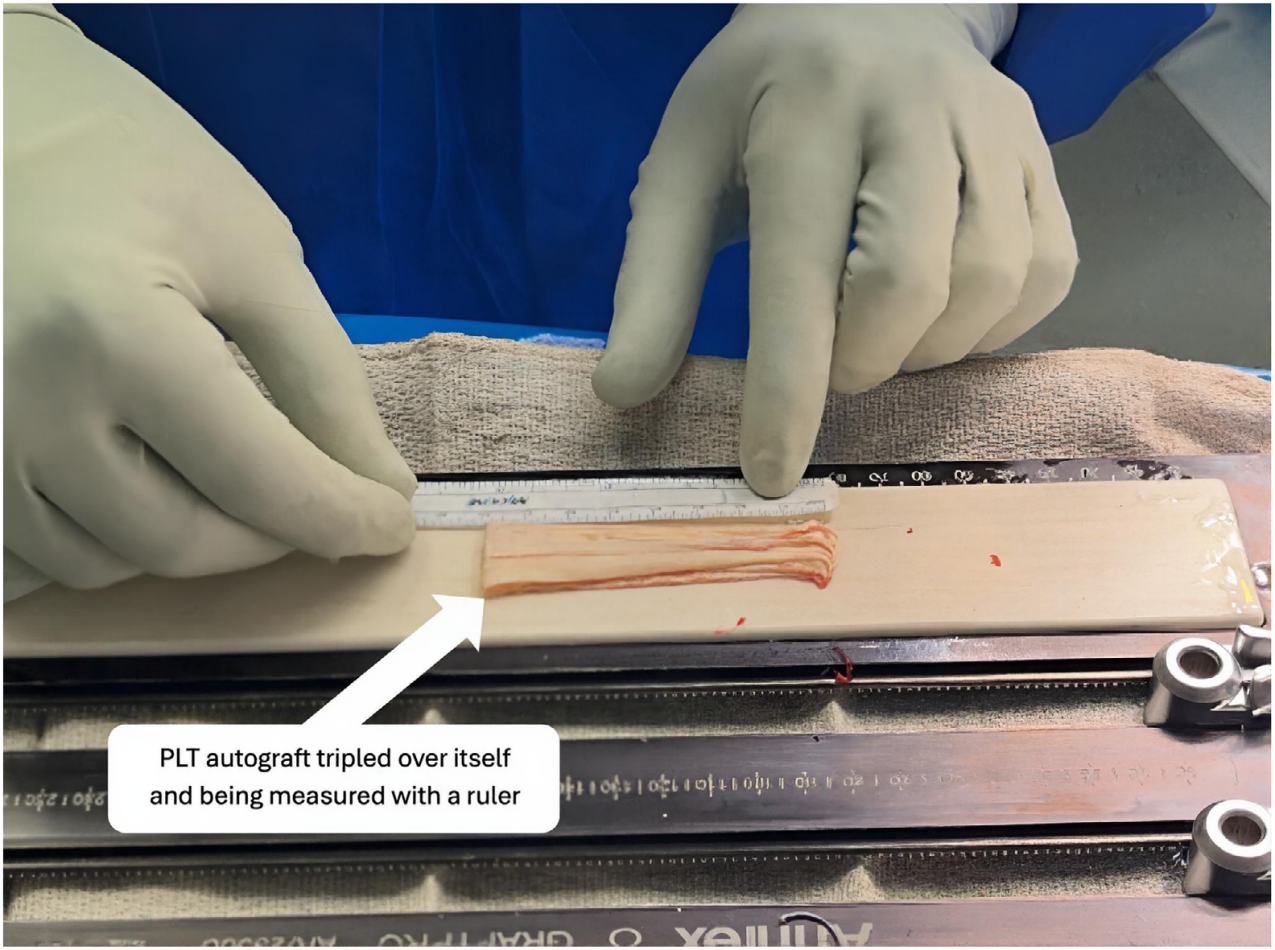
Measurement of peroneus longus autograft. The harvested tendon measures 270 mm. The graft is then tripled over itself, and a ruler is used to confirm the peroneus tendon graft length. (PLT, peroneus longus tendon.)

**FIGURE 6:**
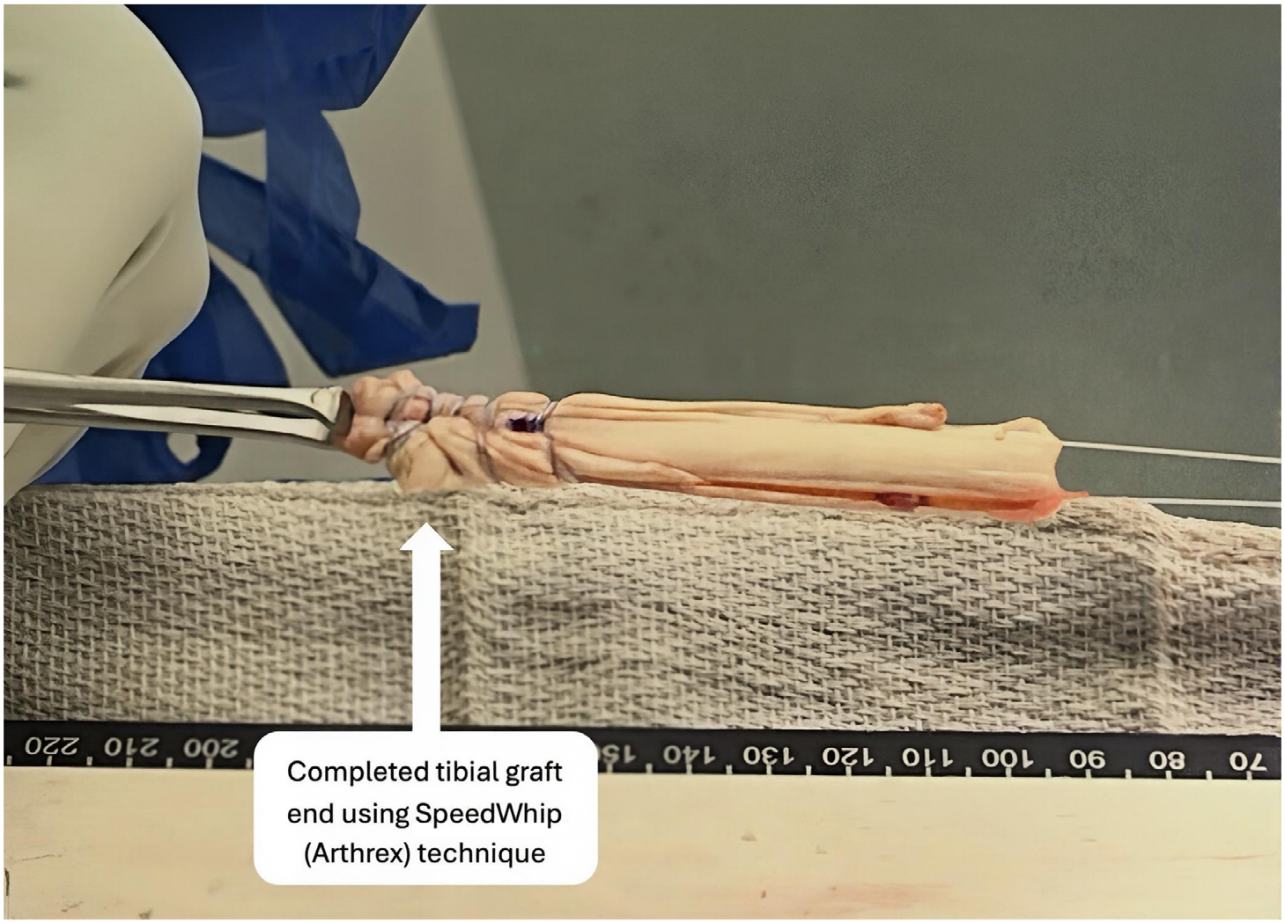
Tibial end preparation of peroneus longus autograft. The tibial end of the graft is secured using 1.3‐mm FiberLoop SutureTape whipstitch (Arthrex) with SpeedWhip (Arthrex) technique. Approximately 20 mm of the distal graft end is incorporated into the whipstitch to allow for reliable tibial fixation and controlled tensioning during reconstruction.

**FIGURE 7:**
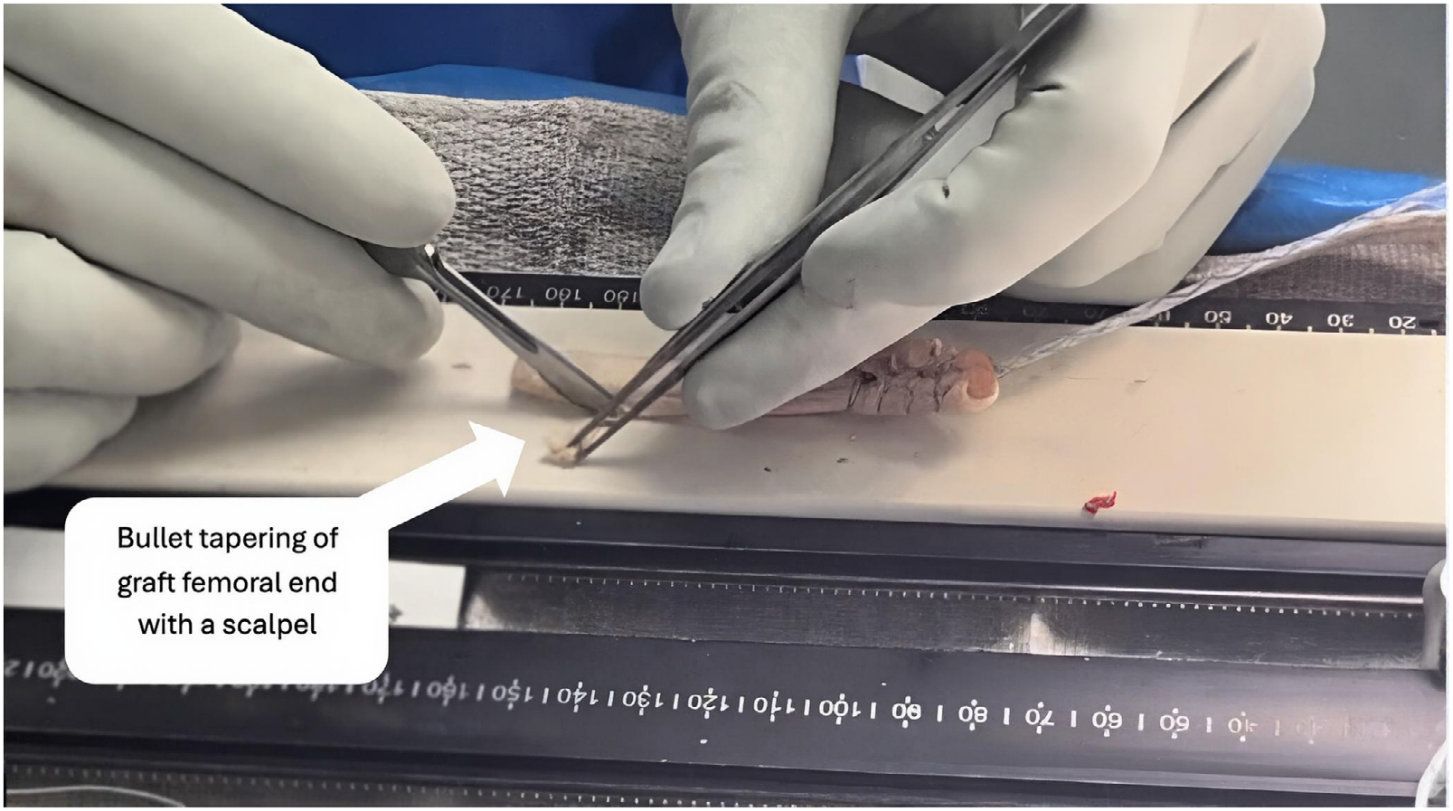
Femoral end bullet tapering of peroneus longus autograft. Preparation of the femoral side begins with bullet‐tapering the free ends of the graft with a scalpel to facilitate atraumatic passage and seating within the femoral tunnel. As shown in the image, a scalpel is used to taper the ends of the femoral side of the graft to the surgeon's preference.

**FIGURE 8:**
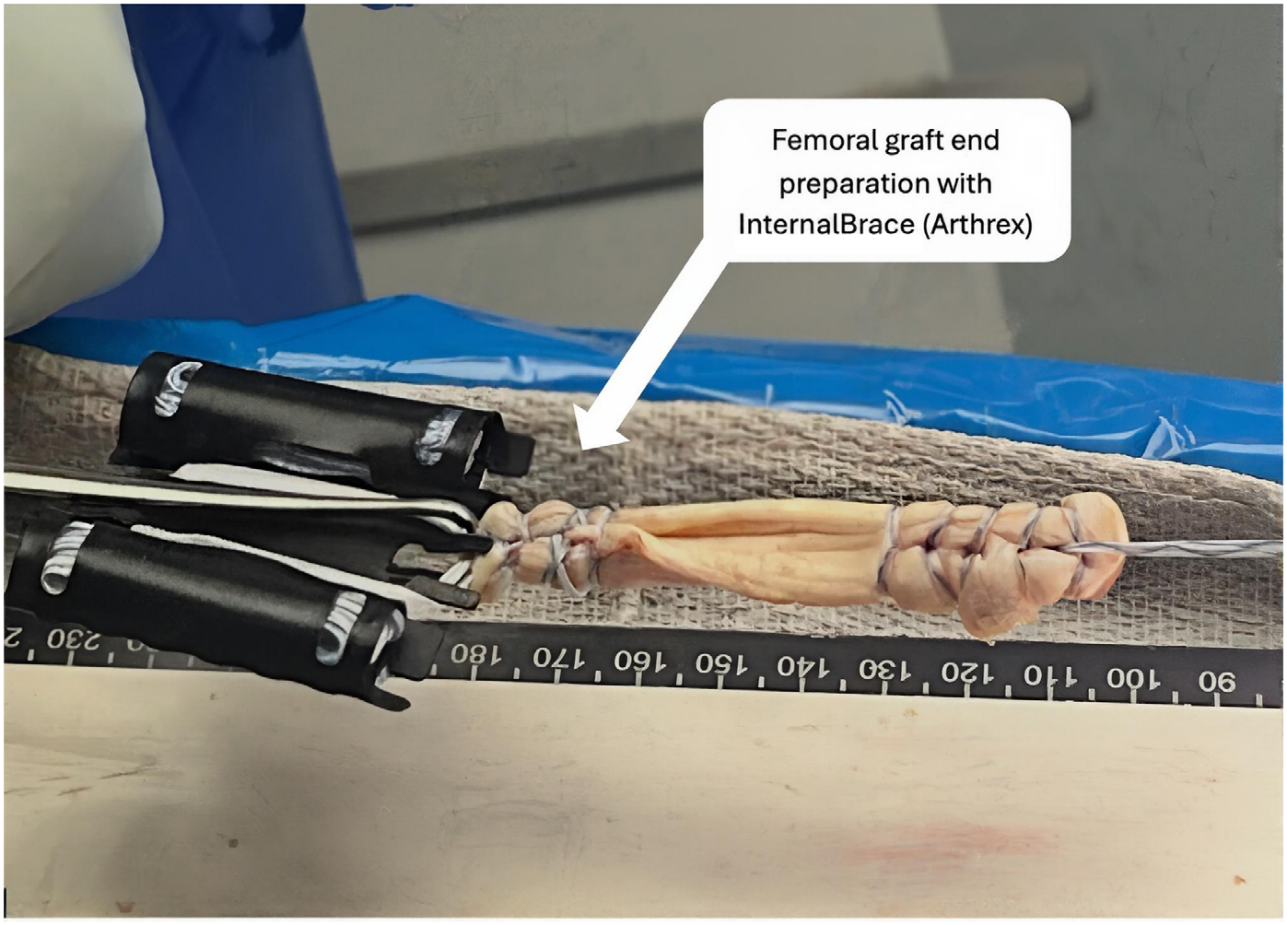
Femoral end preparation of peroneus longus autograft. Femoral fixation is achieved by attaching a FiberTag TightRope II Implant (Arthrex) with InternalBrace (Arthrex) to the proximal graft end, again incorporating 20 mm of graft within the fixation sutures. The InternalBrace (Arthrex) is replaced with Zimmer ActivBraid Collagen Co‐Braid internal brace (Zimmer Biomet) as it adds additional collagen to the graft construct as a biointegrative high‐strength collagen suture.

**FIGURE 9:**
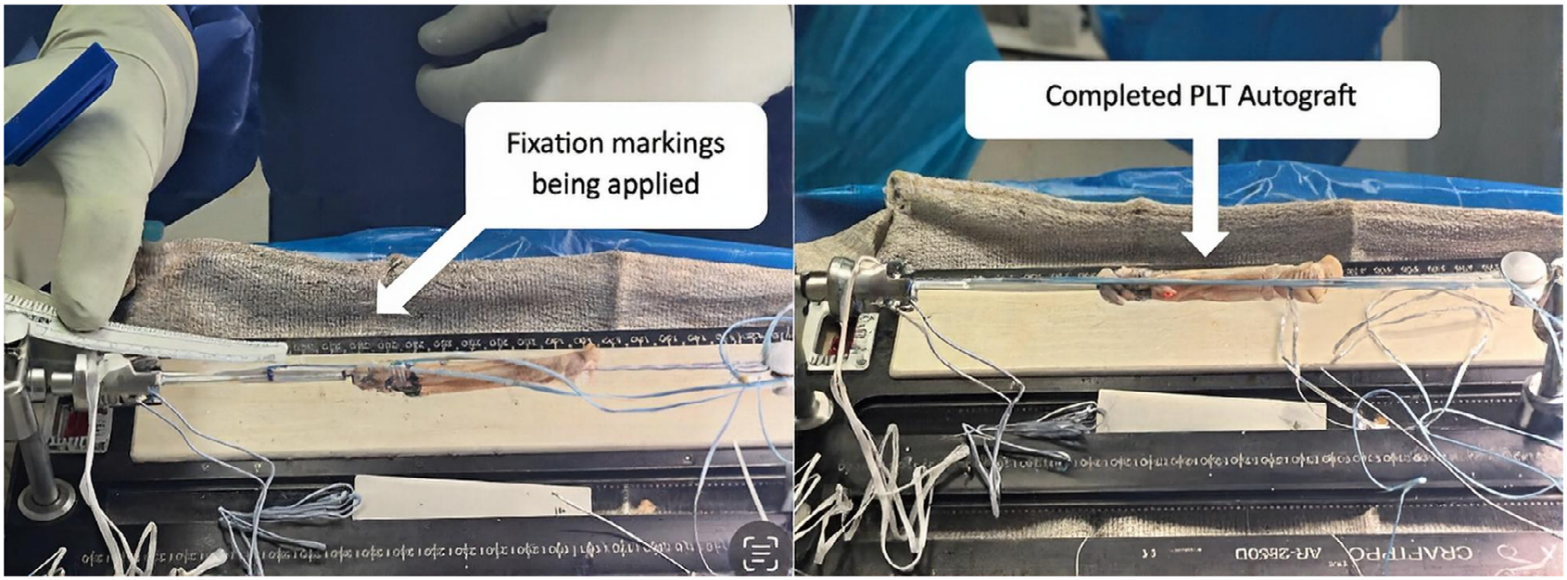
Completion of peroneus longus autograft. This figure shows the completion of the PLT autograft preparation after fixation markings have been marked for proper tunnel seating. This graft will be used for ACL reconstruction. (ACL, anterior cruciate ligament; PLT, peroneus longus tendon.)

A demonstration of this technique in a left knee with an ipsilateral PLT autograft harvest and preparation is provided in Video 1. Figures 1, 2, 3 to 4 depict PLT graft harvest. Figure 5 shows graft harvest measurement. Figure 6 shows completion of the tibial end, while Figures 7 and 8 show bullet tapering and completion of the femoral end of the graft, respectively. Figure 9 shows the product of PTL autograft preparation. The advantages and disadvantages are described in Table 1, while the pearls and pitfalls are summarized in Table 2.

**TABLE 1:** Advantages and Disadvantages

Advantages
• High tensile strength
• Consistent graft diameter with predictable length
• Compatible with suspensory fixation and internal brace augmentation

Disadvantages
• Limited long‐term clinical outcome data
• Unfamiliar peroneal tendon harvest instructions meaning a learning curve is required
• Requires additional care to avoid sural and common peroneal nerve injury during harvest

**TABLE 2:** Pearls and Pitfalls

Pearls
• Measure full tendon length immediately after harvest before graft preparation
• Tripling over peroneal tendon graft to ensure uniform diameter and improved structural integrity
• Use standardized markings to ensure proper button flip
• Bullet‐tapering the femoral end improves atraumatic passage

Pitfalls
• Inadequate graft measurement may lead to insufficient length for tripling
• Uneven tensioning during tripling can create nonuniform graft diameter
• Failure to mark may result in improper seating, soft‐tissue interposition, or button malposition
• Over‐tapering may comprise graft bulk

### Peroneus Longus Tendon Graft Harvest

For harvest of the peroneus longus, a key anatomical landmark is the distal fibula. The peroneus tendons run just posterior to the distal fibula, so this is marked with a skin marker (Figure 1). Additionally, a mark is placed 5 cm down from the proximal fibular head to indicate the most proximal extent for tendon stripping and harvest. A 2‐ to 3‐cm incision is made just proximal to the tip of the distal fibula and approximately 1 cm posterior to the posterior border of the fibula.^7^ The skin and soft tissue is sharply incised, revealing the fascia overlying the peroneal tendons to be removed. Just proximal to the posterior fibular border, the peroneus brevis may be identified by having a low‐lying muscle belly, while the longus is tendinous at this location. An Acufex Smith and Nephew Curved Hemostat (Acufex Smith and Nephew, Los Angeles, CA) is then passed deep to the peroneus brevis, bringing both tendons outside of the skin to allow for suture tenodesis of distal longus to brevis. A #0 FiberWire Suture (Arthrex, Naples, FL) is used for tenodesis of the longus to brevis just proximal to the tip of the distal fibula (Figure 2) and to hold tension for tendon harvest. The Acufex Smith and Nephew Curved Hemostat (Acufex Smith and Nephew, Los Angeles, CA) is then placed between the longus and brevis, followed by a running 1.3‐mm FiberLoop SutureTape whipstitch (Arthrex, Naples, FL) starting 3 to 4 cm proximal to the previously placed tenodesis suture and working distal toward the tenodesis site. This whipstitch suture allows for control of the longus during tendon harvest.

Once the 1.3‐mm FiberLoop SutureTape whipstitch (Arthrex, Naples, FL) has been placed, the peroneus longus is transected with a scalpel just proximal to the tenodesis suture and distal to the whipstitch suture. The transected end of the peroneus longus is controlled by the whipstitch sutures and placed through an Acufex Smith and Nephew Tendon Stripper Open (Acufex, Los Angeles, CA) (Figure 3). An open or closed tendon stripper is used depending on surgeon preference. The tendon is then stripped proximally while keeping the Tendon Stripper Open (Acufex Smith and Nephew) parallel to the fibula and successfully harvested (Figure 4).^8^ It should be noted that the Tendon Stripper Open (Acufex Smith and Nephew) should stop at least 5 cm distal to the fibular head using the skin marking made prior to incision to avoid injury to the deep peroneal nerve.^3^, ^6^, ^7^, ^8^ From previous experience, the tendon always releases 10 cm or more distal to the fibular head.

### Peroneus Longus Tendon Graft Preparation

After harvest of the PLT, the total graft length is measured on a GraftPro AR‐2950D Preparation Board from the Arthrex GraftPro Graft Preparation Set (Arthrex, Naples, FL). In general, the transected PLT is often 270 mm or longer and can be either doubled or tripled over based on surgeon size and length preference. As shown in Figure 5, a ruler is used to confirm the peroneus tendon graft length. When tripled, the graft measured approximately 10 mm in diameter.^7^, ^8^ The tripled graft diameter is sized using a Graft Sizing Block AR‐1886 (Arthrex, Naples, FL) to confirm a uniform diameter of 10 mm along its entire length. Once appropriate sizing is verified, attention is directed to the preparation of the tibial and femoral ends.

### Tibial and Femoral End Preparation

The tibial end of the graft is secured using a 1.3‐mm FiberLoop SutureTape whipstitch (Arthrex, Naples, FL) with standard SpeedWhip (Arthrex, Naples, FL) technique as shown in Figure 6. Approximately 20 mm of the distal graft end is incorporated into the whipstitch to allow for reliable tibial fixation and controlled tensioning during reconstruction.

Preparation of the femoral side begins with bullet‐tapering the free ends of the graft to facilitate atraumatic passage and seating within the femoral tunnel which has a diameter of 10 mm (Figure 7). Femoral fixation is achieved by attaching a FiberTag TightRope II Implant (Arthrex, Naples, FL) with InternalBrace (Arthrex, Naples, FL) to the proximal graft end, again incorporating 20 mm of graft within the fixation sutures (Figure 8). This technique allows for suspensory fixation and controlled cortical button deployment.

The InternalBrace (Arthrex, Naples, FL) component is removed and replaced with a Zimmer ActivBraid Collagen Co‐Braid internal brace (Zimmer Biomet, Warsaw, IN), after the Zimmer ActivBraid Collagen Co‐Braid internal brace (Zimmer Biomet, Warsaw, IN) became available at our institution, because it adds additional collagen to the graft construct as a biointegrative high‐strength collagen suture. Either brace can be used based on surgeon preference.

### Fixation Markings for Tunnel Seating

To assist with proper button flipping and avoidance of iliotibial band incorporation during femoral tunnel deployment, the tightrope is marked at 38 mm from the button, corresponding to the measured femoral cortical thickness. Our technique is most commonly with a full tibial tunnel, and fixation is with a biocomposite interference screw in the tibial tunnel. A second mark is placed 20 mm from the femoral graft end, serving as a visual indicator to confirm full graft seating within the femoral socket during final fixation. The femoral fixation is a tightrope suspensory fixation in the femoral socket. The final graft product is shown in Figure 9.

## DISCUSSION

The use of the PLT as an autograft for ACL reconstruction has gained increasing interest because of its reliable diameter, favorable biomechanical characteristics, and low donor‐site morbidity.^7^, ^8^ Prior studies show that the PLT possesses tensile strength comparable to or exceeding native ACL strength and is biomechanically similar to semitendinosus and gracilis grafts.^8^, ^9^ Clinical outcomes also appear encouraging, with authors reporting preserved ankle strength, low‐harvest site complications, and functional results equivalent to hamstring grafts.^9^ Despite this, PLT graft use remains relatively under‐reported in the United States literature, particularly regarding technical nuances of graft preparation.

Our report adds to the growing body of evidence by offering a detailed, stepwise description of PLT preparation, emphasizing features that enhance reproducibility and intraoperative efficiency. Tripling the graft creates a durable construct measuring approximately 10 mm in diameter. Internal brace augmentation is used to enhance early mechanical stability and potentially reduce elongation during the graft healing phase.^10^ In this technique, integration of a collagen co‐braid brace adds additional collagen to the graft construct; however, either brace types can be used based on surgeon preference. A standardized marking strategy is used to optimize femoral tunnel seating and prevent soft‐tissue interposition. These details may meaningfully affect the consistency of graft placement.

As interest in PLT autografts grows, broader reporting of harvest and preparation strategies will help refine best practices and clarify indications for which patient populations may benefit most from this graft option.

## DISCLOSURES

The authors (M.P., N.B., T.R., K.H., T.M.) declare that they have no known competing financial interests or personal relationships that could have appeared to influence the work reported in this article.
